# Targeting the Reactive Proteome: Recent Advances in Activity-Based Protein Profiling and Probe Design

**DOI:** 10.3390/biom15121699

**Published:** 2025-12-05

**Authors:** Yuan-Fei Zhou, Ling Zhang, Zhuoyi L. Niu, Zhipeng A. Wang

**Affiliations:** Desai Sethi Urology Institute & Sylvester Comprehensive Cancer Center, University of Miami Miller School of Medicine, Miami, FL 33136, USA; yxz2215@med.miami.edu (Y.-F.Z.); lzhang@med.miami.edu (L.Z.); zxn90@miami.edu (Z.L.N.)

**Keywords:** reactive proteome, ABPP, probe design, chemical biology

## Abstract

Activity-based protein profiling (ABPP) has emerged as a powerful chemical proteomics approach for profiling active amino acid residues, mapping functional proteins, and guiding covalent drug development in complex biological systems. Recent methodological advances have produced several novel formats, including tandem orthogonal proteolysis-ABPP (TOP-ABPP), isotopic tandem orthogonal proteolysis-ABPP (IsoTOP-ABPP), and competitive IsoTOP-ABPP, enabling broader target identification and quantitative analysis for varied experimental purposes. In parallel, chemical probe design has evolved to selectively target specific amino acid residues, such as cysteine (Cys), lysine (Lys), and histidine (His), and to incorporate photoaffinity labeling (PAL) functionalities for capturing transient or weak protein-ligand interactions. Additionally, the integration of cleavable linkers with diverse cleavage mechanisms, including acid/base-mediated, redox-mediated, and photo irradiation mechanisms, has enhanced probe versatility and downstream analytical workflows. This review summarizes recent advances in ABPP methodologies and the design of activity-based probes and PAL probes, emphasizing their implications for future work in chemical biology.

## 1. Introduction

Amino acid residues, as the fundamental building blocks of proteins, determine protein functions not only through their abundance but also through their intrinsic chemical reactivity. Even residues of the same type can exhibit distinct reactivities depending on their primary sequence, local pH and hydrophobicity, solvent accessibility, and neighboring catalytic or electrostatic environments. These complex microenvironmental factors endow specific residues within protein-active sites with unique chemical properties, enabling catalytic activity, redox sensing, metal coordination, ligand recognition, and post-translational regulation [1].

Unlike traditional biochemistry and molecular biology approaches that study the structure and activity of proteins one molecule at a time, the ability to distinguish the activity levels of amino acid residues across thousands of different proteins at the omics scale has opened a new pathway to understanding the reactive proteome by chemical proteomics. This approach is especially powerful for dissecting complex protein networks under physiological conditions, uncovering disease mechanisms in pathological states, and guiding the rational design of covalent drugs [2,3].

Against this backdrop, activity-based protein profiling (ABPP) originated from enzyme-class probes and transformed into a general platform for mapping the reactive proteome, using modular probes (warhead-linker-reporter) to covalently capture functional residues under native, selective, and spatiotemporally defined conditions [4,5]. Advances in mass spectrometry, bioorthogonal chemistry, and enrichment strategies have transformed ABPP from gel-based detection to site-resolved, quantitative proteomics, exemplified by tandem orthogonal proteolysis (TOP)-ABPP for on-bead site release and Isotopic (iso)TOP-/Tandem Mass Tag (TMT)-/Stable Isotope Labeling by amino acids in Cell Culture (SILAC)-ABPP for multiplexed quantification and competition assays [6,7].

Concurrently, warhead chemistry has diversified beyond classical cysteine (Cys) alkylation to encompass lysine (Lys), histidine (His), methionine (Met), tyrosine (Tyr), tryptophan (Trp), etc. [8,9]. With the introduction of new families of warhead derivatives into the ABPP field, distinct subsets of the same amino acid residues can now be profiled for their unique activities, providing valuable guidance for covalent drug development. Additionally, photoaffinity labeling (PAL) complements ABPP by capturing non-covalent interactions in situ through photoreactive groups such as diazirines, benzophenones, aryl azides, and other newly developed tags that fine-tune radical lifetimes and off-target reactivity. This advancement expands ABPP beyond covalent chemistry, enabling the systematic exploration of protein-protein and protein-ligand interactions mediated by non-covalent binding.

Finally, cleavable linkers enable stringent washing and controlled release for confident site identification. This principle underlies the core concept of “tandem” in TOP-ABPP, where target enrichment occurs twice, first at the protein level and then at the peptide level. Such dual enrichment effectively removes background contaminants and allows high-fidelity identification of modification sites.

In this review, we summarize recent conceptual and practical advances in ABPP across three major aspects: (1) platform enhancement enabling quantitative proteomic analysis, (2) residue-expansive strategies that extend site coverage, and (3) linker engineering that enables robust enrichment and accurate readouts. We also conclude with a perspective on current limitations and future opportunities for translating reactive-proteome maps into mechanism-driven biology and covalent drug development.

## 2. ABPP and Its Advanced Platforms

As a pioneer in the field of ABPP, Dr. Benjamin F. Cravatt has played a foundational role in shaping nearly every stage of modern chemoproteomic strategy development, from basic ABPP to advanced platforms such as TOP-ABPP and IsoTOP-ABPP. Virtually all existing ABPP workflows trace their conceptual and technical origins back to his laboratory. To acknowledge this legacy and provide clarity, we introduce several representative ABPP platforms in this section, highlighting key methodological innovations and potential applications.

### 2.1. Basic Form of ABPP

**Figure 1:**
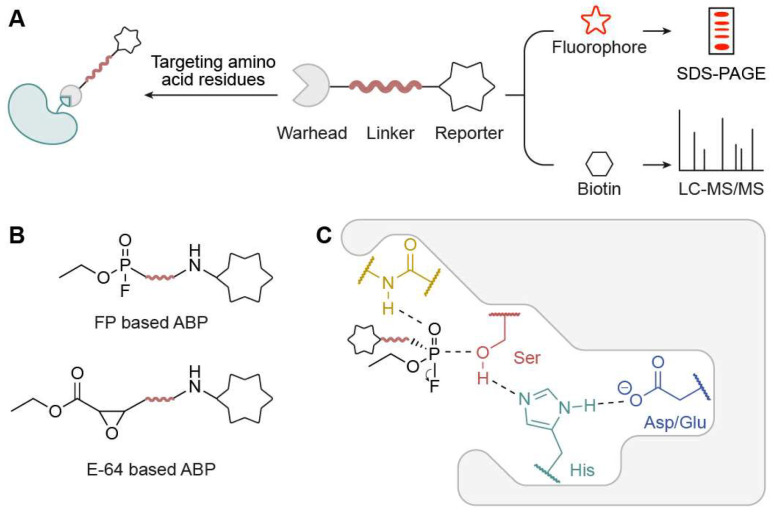
Overview of activity-based probes (ABPs). (**A**) A typical ABP bears a reactive group (warhead) and a reporter group via a connecting linker. (**B**) Representative ABPs include FP-based probes and E-64-based probes. (**C**) Illustration of ABP engagement with a representative serine hydrolase active site.

ABPP is an emerging targeted proteomics platform developed by Benjamin Cravatt and Matthew Bogyo that employs chemical probes that are directed towards specific sites to determine the active states of many enzymes [10,11]. In ABPP, activity-based probes (ABPs) detect the active status of a protein under certain spatiotemporal conditions rather than its total abundance. An ABP (Figure 1A), which normally consists of a reactive group, a reporter group, and a connecting linker, is designed and synthesized in ABPP. The reactive groups covalently bond to nucleophilic amino acid residues in target proteins, typically located within active sites and often involving key catalytic residues. The reporter group, such as fluorophore or biotin, can record ABP labeling on target proteins for follow-up detection. When connected to fluorescent groups like luciferin, rhodamine, or Cy3/Cy5, the labeled protein will exhibit distinct fluorescence signals, enabling easy detection when the proteome identified by the probe is separated on SDS-PAGE, a process known as an “in-gel fluorescence” assay. In the case where the probe employs biotin as the reporter group, these proteins can be enriched by streptavidin, digested by trypsin, and then ascertained via mass-spectrometry analysis. The most representative ABPs include the fluorophosphonate (FP)-based probe developed by the Cravatt lab, which targets serine hydrolases, and the epoxide (E-64)-based probe from the Bogyo lab, which targets Cys proteases (Figure 1B). The FP-based probe covalently modifies the active-site serine residue in serine hydrolases, with stabilization provided by interactions within the catalytic triad (Ser-His-Asp/Glu), exemplifying the mechanism of ABP engagement in a prototypical hydrolase enzyme (Figure 1C).

### 2.2. TOP-ABPP

In ABPP, trypsin digestion leaves labeled peptides on the resin, resulting in a loss of site-specific information. This systematic limitation has long constrained the precision of protein chemical and biological studies. With the development of MS instruments, advanced chromatography tools, and click chemistry techniques [12,13], a TOP-ABPP strategy was developed for the identification of both proteins and their labeling sites [6]. The TOP-ABPP approach integrates click chemistry, biotin-streptavidin enrichment, and the TOP strategy to selectively capture probe-labeled proteins, sequentially releasing unmodified peptides and subsequently modified peptides for liquid chromatography coupled with high-resolution tandem mass spectrometry (LC-MS/MS) analysis. The tandemly regulated release mechanism in TOP-ABPP, in which the labeled peptide remains covalently bound to the resin during trypsin digestion and is released only after subsequent orthogonal cleavage, provides another major advantage. In contrast, antibody-based proteomics allows only a single enrichment step, as trypsinization causes immediate elution from the beads. This dual enrichment in TOP-ABPP markedly enhances specificity and confidence in labeling site identification.

**Figure 2:**
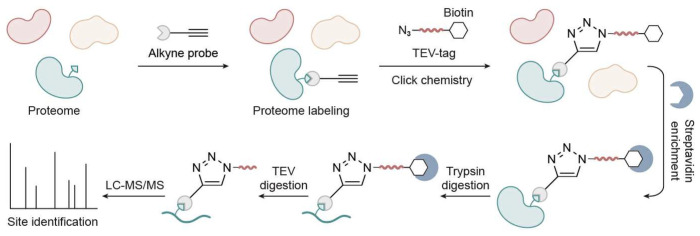
Schematic workflow of TEV-cleavable tag-based TOP-ABPP.

In brief, the proteome is first labeled with an alkyne-containing probe (instead of a fluorophore or biotin) (Figure 2), which selectively reacts with target residues. A copper-catalyzed azide-alkyne cycloaddition (CuAAC) is then used to attach a Tobacco Etch Virus (TEV)-biotin tag to the labeled proteins. This tag includes an azide group, a biotin moiety for affinity enrichment, a flexible linker, and a specific seven-amino-acid recognition sequence cleavable by TEV protease. Following labeling, the biotinylated proteins are captured using streptavidin beads. The immobilized proteins are then reduced and alkylated to prevent disulfide bond formation and stabilize Cys residues. On-bead digestion with trypsin releases non-biotinylated peptides, which are collected via filtration and reserved for LC-MS/MS analysis. To specifically release probe-labeled peptides, the beads are subsequently treated with TEV protease, which cleaves precisely between the glutamine (Gln) and glycine (Gly) residues within its recognition sequence (ENLYFQ↓G). This step ensures that only peptides carrying the chemical probe are released into solution, while the bulky biotin tag remains bound to the beads. The resulting solution is then analyzed via LC-MS/MS to identify probe-modified sites with high specificity.

In most cases, an azide tag is used to trigger a click reaction with an alkyne-containing probe. When the alkyne group is unstable or unsuitable under capturing conditions, a reverse probe bearing an azide moiety can be employed to react with an alkyne tag instead [14]. In addition, strain-promoted azide-alkyne cycloaddition (SPAAC) and inverse-electron-demand Diels-Alder (IEDDA) reactions are widely used clickable handles for bioorthogonal labeling in live cells and in vivo [15,16].

### 2.3. IsoTOP-ABPP and Competitive IsoTOP-ABPP

**Figure 3:**
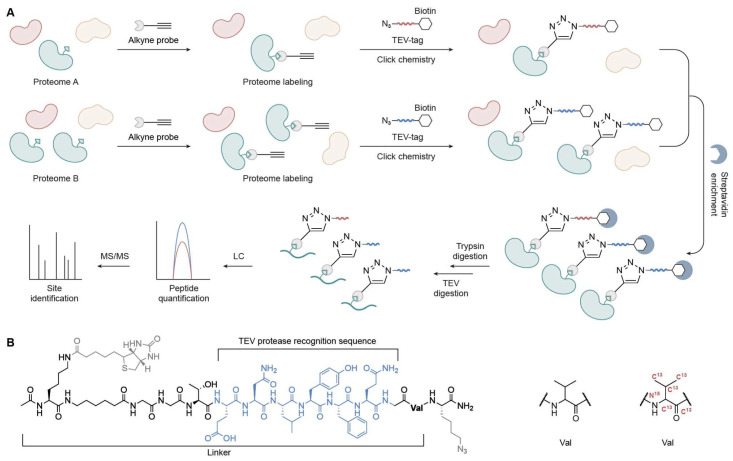
Quantitative TEV-based TOP-ABPP workflow. (**A**) Schematic overview. (**B**) Structure of the TEV-cleavable biotin tag showing the TEV protease recognition sequence and isotopically labeled valine for quantification.

To enable the quantification of the intrinsic reactivity of amino acid residues such as Cys and achieve relative protein quantification across proteomes, the IsoTOP-ABPP method was developed [17]. This strategy leverages quantitative mass spectrometry and offers a key advantage over TOP-ABPP by incorporating isotopically labeled residues into the TEV-tagged linker, such as uniformly ^13^C and ^15^N-labeled valine residues. This design ensures identical chromatographic retention between the light and heavy labeled peptide pairs, while a +6 Da mass difference is sufficient to enable accurate MS-based distinction and quantification (Figure 3).

In brief, the two proteomes of interest (A and B) are reacted with alkyne probes and labeled with light and heavy isotopes, respectively. After enrichment, the labeled proteomes are digested sequentially with trypsin and TEV protease. The probe-labeled peptides, now bearing isotopic tags, are specifically released and collected. These peptides are then analyzed via LC-MS/MS. Quantification is performed based on MS1 and MS2 spectra, and an R ratio is calculated for each active Cys captured by the alkyne probes. This ratio reflects the relative signal intensities of light versus heavy isotopic tags, thereby indicating differences in Cys reactivity across conditions.

IsoTOP-ABPP can be adapted to a competitive format for proteome-wide identification and quantification of Cys interactions with electrophilic small molecules, a process termed competitive IsoTOP-ABPP [18]. As a representative example, proteome-wide reactivity toward lipid-derived electrophiles (LDEs) can be systematically profiled via pre-treatment with either an LDE or DMSO as a control. Subsequently, both samples are labeled with an iodoacetamide-alkyne (IA-alkyne) probe and conjugated to isotopically distinct TEV tags, either light or heavy, respectively, via CuAAC. After equal amounts of the light (control) and heavy (treated) proteomes are mixed, peptide enrichment, TEV protease cleavage, and mass-spectrometry-based identification are performed, as described in the IsoTOP-ABPP workflow. Labeled Cys-containing peptides are analyzed via LC-MS/MS, and the relative labeling is quantified using the MS1 chromatographic peak intensities. The resulting R value, calculated as the ratio of light (control)-to-heavy signal intensities, reflects the degree of competition. A high R value indicates reduced IA-alkyne probe labeling upon LDE treatment, suggesting that the corresponding Cys is highly reactive and sensitive to electrophilic modification by LDEs.

### 2.4. Other Platforms

Other platforms of ABPP involve the development of quantitative strategies, such as rdTOP-ABPP [19], TMT-ABPP [7], SILAC-ABPP [20], and DIA-ABPP [21]. For instance, TMTs have been integrated into ABPP workflows to enable MS2-based quantification. Cravatt and colleagues developed TMT-ABPP to profile druggable cysteines in primary human T cells [7]. Another widely used approach is SILAC-ABPP, in which cells are cultured in isotopically light or heavy amino acid media, allowing complete incorporation of labeled amino acids into the proteome for accurate quantitative comparison [20]. In addition, various isotopically cleavable linkers (see Part 4), such as Dialkoxydiphenylsilane (DADPS), have been designed to facilitate quantification during MS analysis [22].

## 3. Design of Reactive Warheads

The design and synthesis of high-quality ABPs are critical for the success of ABPP [23]. Most ABPs share a common modular architecture consisting of a reactive group or warhead, a linker, and a reporter tag. Reporter groups such as fluorescent dyes or biotin are commonly used to enable visualization or enrichment of labeled proteins for downstream analysis. The linker, typically hydrophilic, lipophilic, or peptide-based, serves to spatially separate the reactive and reporter groups, minimizing steric hindrance and improving probe performance (see Part 4). Reactive groups are typically derived from either known covalent inhibitors of the target enzyme or functional moieties with selective reactivity toward specific amino acid residues derived from protein chemistry and bioorthogonal chemistry.

Warheads are particularly valuable due to their intrinsic electrophilicity, which allows them to covalently target nucleophilic residues such as Cys, Lys, and His [24,25]. The choice of warhead, through variation of its reactive group, can lead to markedly different ABPP outcomes. When evaluating a warhead, two key parameters, reactivity and selectivity, must be considered, as they often act in opposition. Selectivity can be discussed on two levels. The first concerns discrimination across amino acid residues, which is relatively easier to achieve since amino acids possess distinct nucleophilic atoms, conjugated or non-conjugated structures, and varying charge states. In general, broader selectivity across the 20 canonical amino acids is desirable. Warheads with optimized reactivity and residue selectivity are particularly valuable for profiling global proteome reactivity in complex biological systems and for applications such as enzyme activity imaging, quantitative analysis, and target identification.

The second dimension of selectivity involves discrimination of the same amino acid residue. Even when targeting the same amino acid residue, variations in protein folding, local sequence, and microenvironmental factors can give rise to substantial differences in reactivity, producing a continuum that ranges from highly reactive to inert sites. In general, higher intrinsic reactivity often comes at the expense of reduced selectivity, whereas lower reactivity enhances selectivity by labeling only the most reactive residues. This selectivity can be evaluated according to the number of residues modified. For tool probes such as IA-alkyne [18], broad labeling is desirable for capturing as many reactive sites as possible. However, less reactive warheads can also be valuable, as they target distinct subgroups and are often regarded as privileged warheads in medicinal chemistry. Even for highly active warheads, incorporating them into different molecular frameworks to introduce steric hindrance can enhance binding selectivity, further broadening their utility in covalent drug design [26]. This balance between reactivity and selectivity constitutes the central appeal of warhead design. In addition, reversible covalent warheads offer tunable, often less-permanent target engagement and reduced off-target liabilities, as highlighted by recent reviews and exemplar kinase inhibitors [27,28].

### 3.1. Design of Residue-Specific Activity-Based Warheads

#### 3.1.1. Cysteine (Cys)-Specific Warheads

**Figure 4:**
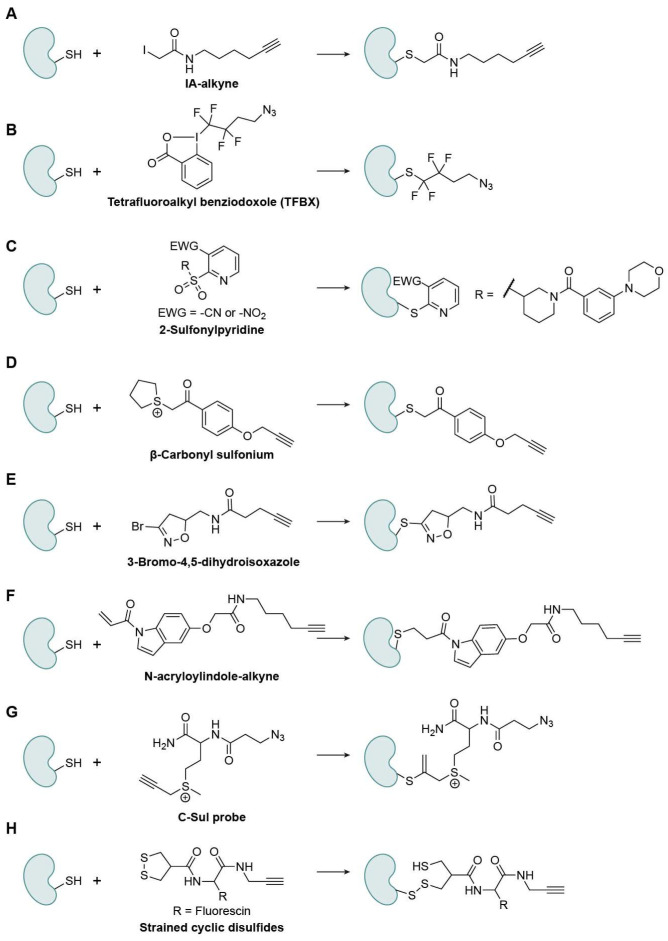
Representative Cys-specific warheads. (**A**) IA-alkyne; (**B**) Tetrafluoroalkyl benziodoxole (TFBX); (**C**) 2-Sulfonylpyridine; (**D**) β-Carbonyl sulfonium; (**E**) 3-Bromo-4,5-dihydroisoxazole; (**F**) N-acryloylindole-alkyne; (**G**) C-Sul probe; (**H**) Strained cyclic disulfides.

Cys is the most intrinsically reactive proteinogenic amino acid because its side-chain thiol (pKa ~ 8–9) is readily deprotonated into a thiolate, the strongest nucleophile in biological environment [29]. Beyond serving as a catalytic nucleophile, Cys residues stabilize protein structure via disulfide formation and metal coordination. Importantly, the reversible disulfide bond formation is strongly affected by the cellular redox environment and can therefore serve as an indicator in cells. In various oxidation statuses, they undergo reversible oxidative modifications (e.g., sulfenylation, S-nitrosylation, and S-glutathionylation), which finely tune redox-sensitive signaling pathways. This chemical versatility makes Cys a prime target for post-translational regulation, covalent drug design, and fragment-based discovery [30,31]. A central goal in this area is to map ligandable Cys proteome-wide with chemoproteomic probes. IA-alkyne was an early ABPP probe that enabled ranking of Cys reactivity across the human proteome (Figure 4A). Since then, diverse electrophiles have expanded coverage and selectivity, considered one of the most active research fields.

##### Nucleophilic-Substitution-Based Warheads

The first class of warheads reacts via a substitution mechanism similar to that of IA-alkyne, where a charged or electron-withdrawing leaving group is replaced by the Cys sulfur through nucleophilic attack [32]. For example, Tetrafluoroalkyl benziodoxole (TFBX) probes from the Adibekian group showed fast kinetics, high chemoselectivity at elevated probe loadings, and improved proteome-wide occupancy relative to IA-Alkyne (Figure 4B) [33]. TFBX probes were further applied in target-ID studies, which reported that XRCC5 was a cellular target of (-)-myrocin G and a simplified analogue. 2-sulfonylpyridines react with Cys residues via a nucleophilic aromatic substitution (S_N_Ar) reaction (Figure 4C) [34]. Tuning electrophilicity and recognition elements yielded a selective covalent modulator of adenosine deaminase that attenuates enzyme activity by engaging a non-catalytic Cys and suppresses lymphocytic proliferation. Li’s lab introduced a novel Cys-targeting electrophile, β-carbonyl sulfonium, which incorporates a β-carbonyl group adjacent to the sulfonium center to enhance electrophilicity at the α-carbon while preserving excellent solubility and stability [35]. This unique design promotes rapid nucleophilic substitution with Cys thiols under physiological conditions, achieving high chemoselectivity over other nucleophiles (Figure 4D). Compared with conventional α-halo carbonyls and iodoacetamide-based probes, β-carbonyl sulfoniums exhibit faster reaction kinetics, a stronger Cys preference, and superior biocompatibility and stability. The optimized probe (CP2) demonstrated efficient labeling of Cys residues in peptides, proteins, and live-cell proteomes, identifying over 1100 Cys sites in MCF-7 cells while also exhibiting minimal cytotoxicity. The tunable sulfonium and β-carbonyl groups enable precise control of reactivity and selectivity, positioning β-carbonyl sulfonium as a next-generation Cys warhead for live-cell ABPP.

Another complementary class, 3-bromo-4,5-dihydroisoxazoles (BDHIs) [36], displays moderate intrinsic reactivity that relies on non-covalent recognition for productive engagement (Figure 4E). Competitive chemoproteomics revealed a selectivity landscape distinct from that for haloacetamides, with effective engagement of reactive cysteines proteome-wide and covalent conjugation to anticancer targets such as Peptidyl-prolyl cis-trans isomerase NIMA-interacting 1 (PIN1) and Glutathione S-transferase P1 (GSTP1). BDHI warheads have also been incorporated via late-stage installation into BTK inhibitor scaffolds, underscoring their synthetic tractability and translational potential.

##### Nucleophilic-Addition-Based Warheads

The second class of warheads reacts through nucleophilic addition, in which a Cys thiol attacks an electrophilic unsaturated bond, forming a covalent adduct. For example, the acrylamide warhead, including maleimide, has long been used as a Michael acceptor that reacts with thiol groups, but it can also form adducts with Lys or His [37]. N-acryloylindoles (NAIs) and their alkyne-tagged variants (NAIAs) place an acrylamide warhead on an indole nitrogen to enhance electrophilicity and cell permeability (Figure 4F) [38]. In lysates and live cells, NAIAs label cysteines more efficiently than IA-Alkyne, enable live-cell imaging of Cys reactivity under oxidative stress, and capture broader Cys subsets in MS-based ABPP, facilitating covalent-ligand screening. Among the hits, CL1, one of the Cys-reactive compounds, engages with Cys178 of Rac family small GTPase 1 (Rac1), suppressing Rac1 signaling and inducing G1 arrest in HepG2 cells. C-Sul probes are a family of sulfonium-based, Cys-reactive probes created by Li’s group designed for live-cell chemoproteomic profiling (Figure 4G) [39]. The probes are water-soluble, stable, and cell-permeable, selectively labeling hyper-reactive cysteines and showing compatibility with quantitative MS while being minimally cytotoxic compared with traditional iodoacetamide probes, enabling in situ mapping of reactive Cys sites in human cells.

##### Disulfide-Bond-Based Warheads

The third class of warheads exploits the unique redox properties of thiol groups, which can undergo reversible oxidation and reduction reactions. Among these, disulfide bonds are particularly useful because they are reversible and easily controlled, enabling selective and tunable covalent interactions under physiological conditions. Adibekian’s lab reported the rational design of a strained cyclic disulfide, asparagusic acid (AspA), as a novel Cys-reactive warhead (Figure 4H) [40]. Unlike free disulfides, the β-thiol ring strain in AspA markedly enhances disulfide exchange reactivity toward surface-exposed Cys residues, providing a tunable and biocompatible approach for cellular entry and Cys-targeted chemoproteomics.

Collectively, the representative warhead families mentioned above exemplify how warhead mechanisms, intrinsic reactivity, and scaffold-driven recognition can be tuned to balance kinetics, chemoselectivity, target occupancy, and live-cell compatibility for mapping and modulating functional Cys [41].

#### 3.1.2. Lysine (Lys)-Specific Warheads

Lys is among the most abundant residues in the human proteome. Because the lysine ε-amino group has a high pKa (~10.5) and is mainly protonated at physiological pH levels, selective covalent modification typically requires one or more of the following: (1) strongly activated electrophiles, (2) local microenvironments that lower pKa or facilitate nucleophilic attack, and/or (3) proximity effects imparted by recognition elements. A broad variety of warheads have been shown to form covalent adducts with Lys in purified proteins, cell lysates, and live cells; these warheads include aryl sulfonyl fluorides, aryl fluorosulfates, Michael acceptors, dichlorotriazines, activated esters/amides, and aryl aldehydes [42].

##### Nucleophilic-Substitution-Based Warheads

**Figure 5:**
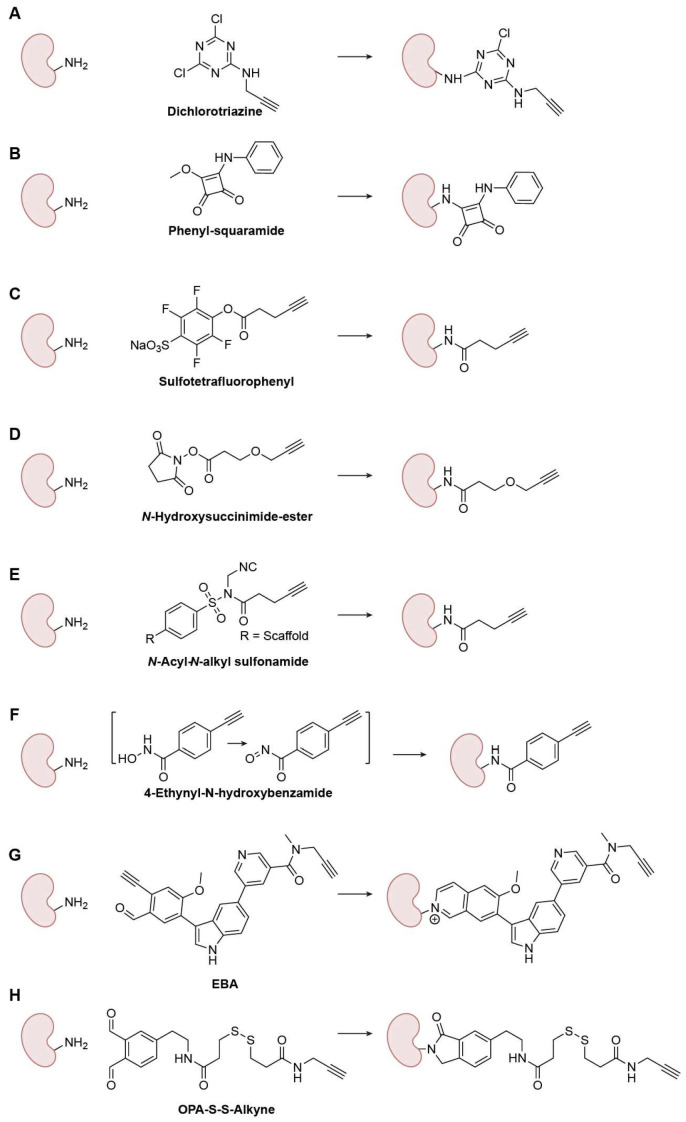
Representative Lys-specific warheads. (**A**) Dichlorotriazine; (**B**) Phenyl-squaramide; (**C**) Sulfotetrafluorophenyl; (**D**) N-Hydroxysuccinimide-ester; (**E**) N-Acyl-N-alkyl sulfonamide; (**F**) 4-Ethynyl-N-hydroxybenzamide; (**G**) EBA; (**H**) OPA-S-S-Alkyne.

Lys amino groups can also participate in nucleophilic substitution reactions; however, their lower nucleophilicity makes achieving selectivity over Cys challenging. Systematic studies from Weerapana’s laboratory evaluated aryl halides operating via SNAr and revealed how ring electronics precisely tune reactivity (Figure 5A) [43]. At low-micromolar probe loadings, dichlorotriazines and *p*-chloro/fluoronitrobenzenes act as covalent protein modifiers. Intriguingly, *p*-chloronitrobenzene favored Cys in complex proteomes, whereas dichlorotriazines were biased toward Lys, representing the first-generation Lys-selective SNAr probes. Squarate chemistry offers another tunable axis [44]. Squarates undergo two sequential conjugate addition–elimination steps with amines; after the first substitution, the resulting monosquaramide displays moderated electrophilicity, slowing the second substitution (Figure 5B). Kiessling and coworkers showed that this kinetic “tempering” favors selectivity for Lys in sustained proximity, whereas highly reactive N-hydroxysuccinimide (NHS) esters react orders of magnitude faster and are correspondingly more promiscuous. Substituent effects and sulfur variants further modulate reaction rates, highlighting how squarates/squaramides and related analogs can be engineered as affinity-compatible handles for chemoproteomic probes.

##### Amide-Bond-Based Warheads

With the advancement of peptide synthesis chemistry, numerous activated acyl reagents have been developed for coupling reactions. Although these reactions remain substitution processes involving Lys amino groups, this family of warheads is highly diverse, encompassing a wide variety of electrophilic scaffolds and mechanisms. Therefore, we consider them distinct from those described in the “Nucleophilic-Substitution-Based Warheads” section. To increase the depth and selectivity of Lys coverage proteome-wide, Cravatt and colleagues introduced the sulfotetrafluorophenyl (STP) ester, which profiles thousands of Lys residues with broader and more Lys-preferential reactivity than aryl-halide S_N_Ar scaffolds (Figure 5C) [45]. Complementarily, Nomura and coworkers designed an alkyne-functionalized NHS ester that served as a versatile reactivity-based probe that maps nucleophilic hotspots (Figure 5D) [46]. By embedding recognition elements, fragment-derived NHS esters achieved target- and site-selective engagement of Lys hotspots on proteins, illustrating that tempered intrinsic reactivity plus binding energy can overcome Lys’s pKa barrier. So far, acyl warheads with varying leaving group activation levels have been developed, each exhibiting diverse selectivity for Lys-targeted profiles [47].

Ligand-directed chemistry further exploits proximity to improve selectivity. Hamachi and colleagues developed N-acyl-N-alkyl sulfonamides (NASA), based on a well-established, highly reactive “safe-catch” linker [48], that undergo rapid acyl transfer to nearby ε-amines under native live-cell conditions [49], enabling labeling of non-catalytic Lys with selectivity and biocompatibility (Figure 5E). Although off-targeting can arise from suboptimal ligands or heterogeneous cellular environments, careful warhead/ligand design coupled with optimized reaction conditions can mitigate these liabilities and enable robust in-cell proximity-driven labeling. Li et al. introduced a novel oxidant-triggered acyl warhead for Lys-targeted ABPP based on a hydroxamic acid scaffold that can be selectively oxidized to generate a carbonyl nitroso electrophile (Figure 5F). This oxidant-triggered bioconjugation platform enables proteome-wide and selective labeling of Lys residues, allowing systematic profiling of their reactivity and ligandability [50]. Using 4-ethynyl-N-hydroxybenzamide, they quantified >7000 covalently modifiable Lys sites and identified >100 endogenous kinases with ligandable catalytic Lys residues, markedly expanding the known Lys ligandability landscape. The cited work also highlighted newly ligandable Lys residues that could serve as potential starting points for targeted covalent inhibitor development.

##### Imine-Based Warheads

Both amino and thiol groups can react with carbonyl compounds to form tetrahedral intermediates. However, a unique feature of Lys amino groups, unlike Cys thiols, is their ability to undergo dehydration to form imines (Schiff bases). Although these imine linkages are reversible, they can be stabilized through subsequent reduction to generate permanent C-N bonds in bioconjugation. For warhead development, reversibility is generally undesirable; therefore, several strategies beyond simple reduction have been developed to enhance Lys selectivity and stabilize the conjugate. Yao’s lab introduced 2-ethynylbenzaldehyde (EBA) as a next-generation Lys-targeting irreversible covalent warhead applicable to both kinases and non-kinases [51]. The EBA scaffold incorporates an intrinsic aldehyde-alkyne motif that reacts with the ε-amino group of Lys to form a stable isoquinolinium adduct, resulting in irreversible C-N bond formation with exceptional amine selectivity (Figure 5G). Unlike conventional sulfonyl fluoride electrophiles, EBA, originally designed for N-terminal modification [52], exhibits balanced reactivity, high hydrolytic stability, minimal glutathione cross-reactivity, and exclusive Lys selectivity under physiological conditions. Together, these properties establish EBA chemistry as a versatile, amine-specific platform for developing Lys-reactive warheads in ABPP and targeted covalent inhibitor design. Lu et al. developed a novel family of di-ortho-phthalaldehyde (DOPA) cross-linkers as highly reactive, non-hydrolyzable Lys-targeting warheads for chemical cross-linking [53]. Each DOPA reagent features two ortho-phthalaldehyde (OPA) groups connected by short ethylene-based spacers, enabling rapid formation of stable isoindolinone linkages with Lys ε-amines or N-terminal amines (Figure 5H). The optimized variant, DOPA2, exhibits high Lys selectivity; fast reactivity even under low-pH, low-temperature, or denaturing conditions; and superior structural fidelity in cross-link mapping. The resulting C-N bonds form irreversibly within seconds, more than one hundred times faster than conventional NHS-esters, making this amine-specific aldehyde warhead a powerful tool for Lys-directed covalent chemistry and dynamic structural studies.

#### 3.1.3. Histidine (His)-Specific Warheads

His imidazole (pKa ~ 6) toggles protonation near physiological pH values, enabling general acid-base catalysis, hydrogen-bonding, metal coordination, and proton shuttling [54].

##### Nucleophilic-Substitution-Based Warheads

**Figure 6:**
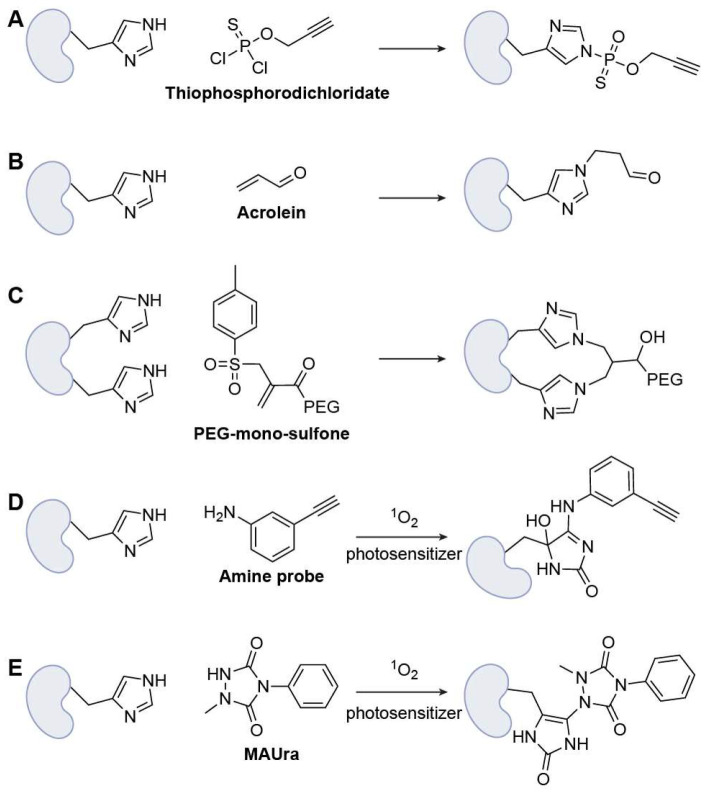
Representative His-specific warheads. (**A**) Thiophosphorodichloridate; (**B**) Acrolein; (**C**) PEG-mono-sulfone; (**D**) Amine probe (with photosensitizer); (**E**) MAUra (with photosensitizer).

The first class of warheads is still based on nucleophilic substitution; however, the moderate nucleophilicity of His makes selective chemoselective labeling challenging in complex proteomes. Chang and co-workers introduced bioinspired thiophosphorodichloridate reagents that mimic His phosphorylation, enabling rapid, selective His labeling under mild conditions and efficient payload installation, with clear utility for His-tag functionalization and live-cell delivery (Figure 6A) [55].

##### Nucleophilic-Addition-Based Warheads

Michael addition remains an important strategy for designing warheads in His-targeted ABPP. Building proteome-scale maps, Ye and colleagues developed an acrolein (ACR)-based probe with reversible hydrazine enrichment to quantify >8200 His in human cells, including 317 hyper-reactive sites, thereby identifying potential His-ligandable hotspots and positioning ACR derivatives as candidate warheads (Figure 6B) [56]. Brocchini et al. introduced a His-reactive bis-alkylation strategy for site-selective protein conjugation, demonstrating that the imidazole group of His can act as a nucleophile in Michael-addition-elimination reactions using PEG-mono-sulfone reagents. Rather than relying on traditional Lys- or Cys-based chemistries, the authors engineered adjacent His residues (His_2_-tags) into defined protein regions while preserving activity, enabling controlled modification under mildly acidic conditions in which His remains nucleophilic while Lys is protonated (Figure 6C) [57]. This work established bis-sulfone alkylation at His_2_ motifs as an effective warhead design for His-directed activity-based protein profiling and therapeutic protein engineering.

##### Oxidation-Based Warheads

Given the structural complexity of the imidazole ring, unique heterocyclic chemistries can be leveraged to achieve bioorthogonal enrichment with high selectivity over other amino acid residues. Recently, Li’s team reported an oxidation-based strategy for His-targeted ABPP employing a singlet-oxygen-driven “relay labeling” chemistry that overcomes the weak nucleophilicity of the His imidazole ring [58]. In this approach, small-molecule photosensitizers generate singlet oxygen under visible light to transiently oxidize the His imidazole, forming a reactive intermediate that is subsequently trapped by an aniline-based probe such as 3-ethynylaniline to yield stable covalent adducts (Figure 6D). This oxidative relay system achieved exceptional selectivity (>97%) for His over other residues, particularly Met, Tyr, and Trp, enabling proteome-wide identification of more than 7000 His sites across over 2400 proteins and revealing numerous previously unannotated active-site and metal-binding His residues. Similarly, 1-methyl-4-aryl-urazole (MAUra) was employed as a labeling reagent together with photocatalysts to achieve residue-specific modification of His through a nucleophilic attack on oxidized imidazole endoperoxides (Figure 6E) [59]. Notably, selectivity could be tuned by modulating the reaction pathway: singlet-oxygen-mediated processes using BODIPY favored His-selective labeling, whereas single-electron transfer (SET) pathways preferentially targeted Tyr residues [60].

#### 3.1.4. Glutamic Acid (Glu)/Aspartic Acid (Asp)-Specific Warheads

Beyond traditional nucleophilic side chains, acidic residues such as Glu and Asp possess carboxyl groups that can serve as electrophilic handles when suitably activated. Recent advances in chemical probe design have enabled selective targeting of these residues, allowing covalent capture of protein carboxylates that were once considered chemically inert. These developments have expanded ABPP into a new dimension, providing tools for mapping the catalytic and structural roles of Glu and Asp across complex proteomes.

**Figure 7:**
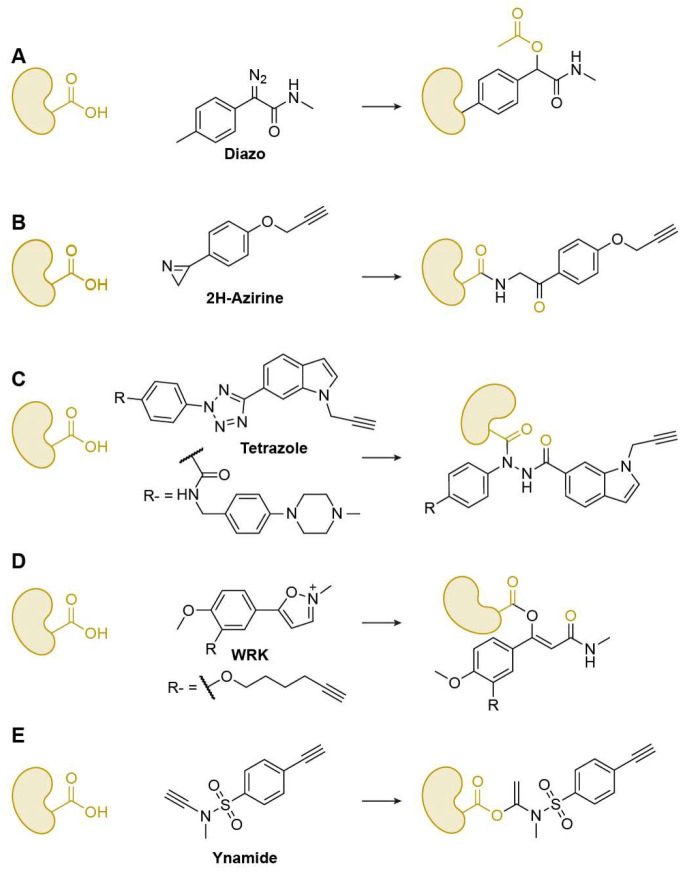
Representative Glu/Asp-specific warheads. (**A**) Diazo; (**B**) 2H-Azirine; (**C**) Tetrazole; (**D**) WRK; (**E**) Ynamide.

Nucleophilic attacks occur at the carboxyl hydroxyl group; however, direct substitution on the carboxyl carbon is generally limited, with diazo compounds being notable exceptions that release N_2_ to form carboxyl esters (Figure 7A). Most other warheads exploit the adjacent carbonyl functionality through tandem reaction mechanisms to achieve effective labeling. In this process, the target carboxyl oxygen first attacks the electrophilic warhead, after which the warhead rearranges and performs a nucleophilic attack back on the carbonyl carbon, forming a stable covalent linkage. Ma and co-workers reported 3-phenyl-2H-azirines as efficient, chemoselective reagents for carboxyl-group modification both in vitro and in cells, enabling quantitative chemoproteomic profiling of reactive carboxyl sites proteome-wide (Figure 7B) [61]. To integrate target identification with phenotypic discovery, Li’s group created a fully functionalized diaryl-tetrazole probe library that supports affinity-based chemoproteomics and live-cell screening; several probes showed potent antiproliferative activity, and the tetrazole probe Tz6 selectively labeled endogenous ANXA2 across mammalian cells (Figure 7C) [62]. Similarly, Hacker developed light-activatable 2,5-disubstituted tetrazoles to map carboxyl residues directly in living Gram-negative bacteria, quantifying 8971 aspartates/glutamates. Using these tools, they introduced hydrazonyl chlorides as a new class of carboxyl-directed covalent ligands [63].

Woodward’s reagent K (WRK), originally developed as a coupling reagent in peptide chemistry, was later adapted into a series of ABPs that exploit a tandem oxygen-carbon attack mechanism to label protein carboxylates with high efficiency [64]. The reactive isoxazolium warhead first undergoes ring opening under basic conditions to generate a ketenimine-like intermediate, which then reacts with the carboxyl oxygen of target residues. The resulting intermediate subsequently rearranges, enabling the warhead enabling the warhead to attack the carbonyl carbon and form a stable covalent linkage. Through this stepwise electrophilic-nucleophilic exchange, WRK-based probes have achieved selective modification of nucleophilic amino acids such as His, Cys, and carboxylates in cell lysates and live cells. These studies have established WRK-inspired (iso)oxazolium chemistry as a versatile framework for developing electrophilic warheads capable of targeting atypical nucleophilic sites in complex proteomes, supporting both proteomic profiling and bioconjugation applications. (Figure 7D) [65].

Beyond the tandem-reaction-based probes described above, many other probes rely on addition-type mechanisms. Examples include those derived from ynamide scaffolds (Figure 7E) and epoxide derivatives, which enable efficient modification of carboxyl groups [66]. Li’s lab systematically evaluated a wide range of these electrophilic scaffolds to advance both covalent drug discovery and proteomic profiling [67].

#### 3.1.5. Warheads Specific to Other Amino Acids

While most ABPP strategies target highly nucleophilic residues such as Cys, Lys, and His, recent advances have extended covalent probe design to less reactive amino acids that play crucial roles in catalysis and signaling. New chemistries exploiting redox activation, sulfur-fluoride or sulfur-triazole exchange, and photo-induced substitution now enable selective modification of Met, Tyr, and Trp under biocompatible conditions, broadening the scope of proteome-wide reactivity profiling.

##### Methionine (Met)-Specific Warheads

**Figure 8:**
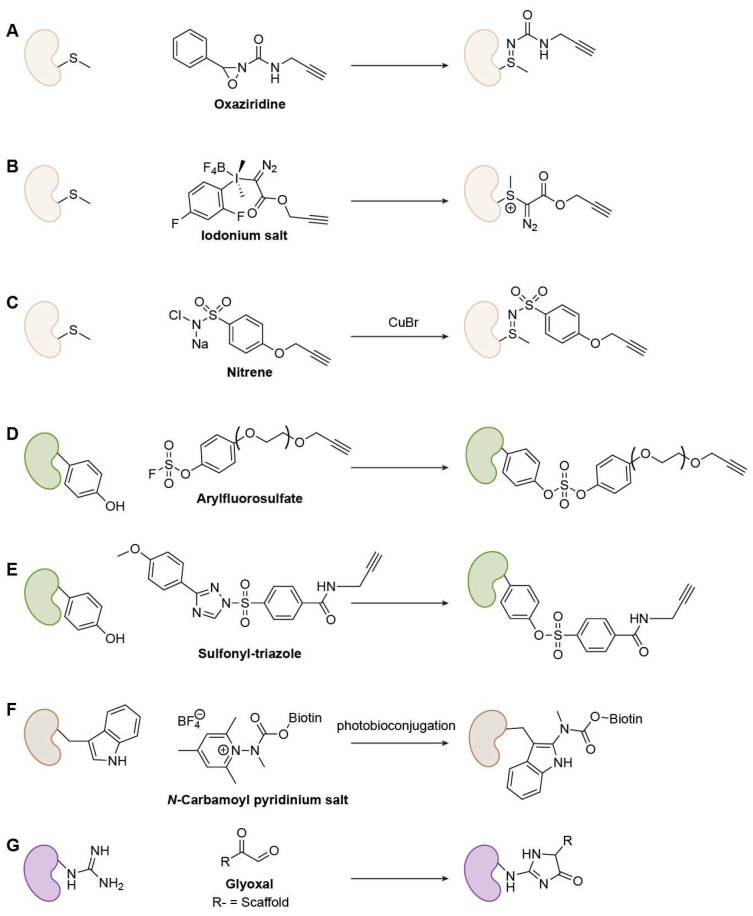
Representative Met-, Tyr-, Trp-, and Arg-specific warheads. For Met: (**A**) Oxaziridine; (**B**) Iodonium salt; (**C**) Nitrene; For Tyr: (**D**) Arylfluorosulfate; (**E**) Sulfonyl-triazole; For Trp: (**F**) N-Carbamoyl pyridinium salt; For Arg: (**G**) Glyoxal.

Met’s thioether is a weak nucleophile, historically limiting selective ligation. Chang and co-workers overcame this using oxaziridine-based redox-activated chemical tagging (ReACT): rapid, chemoselective oxidation of Met to a stable sulfimide enabled robust labeling in biocompatible media, downstream payload installation, and proteome-wide discovery of hyper-reactive Met sites (Figure 8A) [68]. Elsewhere, Gaunt and colleagues developed hypervalent iodine (III) reagents that engage the Met S-Me group via electrophilic activation, delivering fast, low-µM, Met-selective bioconjugation orthogonal to other warheads [69]. The thioether sulfur of Met attacks the iodane electrophile to form a stable cationic sulfonium intermediate, allowing rapid and chemoselective modification under mild, biocompatible conditions (Figure 8B). Raj’s lab developed a Cu(I)-nitrene platform (CuNiP) for chemoselective Met profiling based on a Cu-nitrene mediated sulfimidation mechanism. The Cu-nitrene complex selectively transfers a nitrene group to the Met thioether, forming a stable sulfonyl sulfimide linkage under physiological conditions with high selectivity over other residues (Figure 8C). This strategy enabled proteome-wide identification of more than 600 ligandable Met sites, revealing new targets linked to redox regulation [70].

##### Tyrosine (Tyr)-Specific Warheads

Tyr governs phosphorylation and redox signaling. In this context, chemoselective handles are valuable. Aryl fluorosulfates are less inherently reactive than aryl sulfonyl fluorides, yet within preorganized binding pockets, they react with conserved Tyr residues, highlighting proximity-assisted SuFEx (Figure 8D) [71]. Additionally, Hahm et al. introduced SuTEx (sulfur-triazole exchange): using triazole as a leaving group boosted Tyr chemoselectivity (~5 × over other nucleophiles) and enabled identification of >10,000 Tyr sites from ~3700 proteins in lysates and live cells, including sites in nucleotide-binding, catalytic, and PPI domains (Figure 8E) [72].

##### Tryptophan (Trp)-Specific Warheads

Many Trp warheads suffer from weak selectivity or harsh conditions. Taylor and co-workers addressed this with photobioconjugation using N-carbamoyl pyridinium salts, achieving rapid, aqueous, catalyst- and solvent-free C2-selective Trp functionalization on native biomolecules (Figure 8F) [73]. Even so, protein-compatible Trp covalent platforms remain an active need.

##### Arginine (Arg)-Specific Warheads

Arg is among the most challenging amino acid targets for covalent chemistry due to its strongly basic guanidinium group (pKa = ~12), which remains protonated and poorly nucleophilic under physiological conditions. Two diketone-based chemistries have recently enabled Arg-selective warhead design. Yao et al. developed glyoxal-based Arg-targeting electrophiles that condense with the guanidine group to form stable imidazolidone adducts, achieving irreversible covalent inhibition of both kinases (e.g., AURKA R220) and non-kinases (e.g., Mcl-1 R263) with high site selectivity and cellular activity (Figure 8G) [74]. In parallel, Arg’s guanidinium undergoes nucleophilic addition to 9,10-phenanthrenequinone, forming an imine intermediate that rearranges into glutamate-5-semialdehyde. This reaction converts Arg into a reactive aldehyde, enabling chemoselective profiling of Arg oxidation sites [75]. Together, these advances have established the first generalizable electrophilic and oxidative platforms for Arg-selective ABPP and covalent ligand development.

#### 3.1.6. Outlook for Amino-Acid-Specific Warheads

Hacker et al. [76] compared a set of 56 alkyne probes containing diverse reactive groups. They verified the selectivity of IA-alkyne for Cys, STP-alkyne for Lys, SuTEx alkyne for Tyr, N-carbamoyl pyridinium-alkyne for Trp, and phenyl-2H-azirine for Asp and Glu. However, each probe also exhibited potential off-target reactivity. For example, increasing the concentration of IA-alkyne to 1 mM reduced its Cys selectivity to 86%, which can be compared with the 96% selectivity at 100 µM. Furthermore, STP-alkyne also labeled Ser (9%), Thr (2%), or N-termini (5%). The authors observed Tyr reactivity for the SuTEx alkyne probe was 55–71% and identified Lys residues as the most prominent off-targets (26–41%). The N-carbamoyl pyridinium-alkyne probe showed the expected mass shift of modification in the proteome, showing selectivity for Trp (55%) and His (35%). For the phenyl-2H-azirine probe, 75% of all detected modifications occurred at Asp and Glu, with 18% off-target reactivity at Cys.

Moreover, some warheads have been applied in live-cell studies, including β-carbonyl sulfonium [35] and N-acryloylindoles [37] probes for Cys, N-acyl-N-alkyl sulfonamide for Lys, and phenyl-2H-azirine [61] for Asp and Glu. A more state-of-the-art approach is to use a decaging strategy to enable live-cell Cys chemoproteomics [77]. Furthermore, in vivo ABPP workflows now allow direct measurement of target engagement and off-target effects in intact organisms and tissues [78,79]. Emerging single-cell ABPP and chemoproteomic platforms are pushing activity profiling toward spatially and temporally resolved formats. For example, fluorescent-activity-based probes have been used to phenotype individual *Staphylococcus aureus* cells via microscopy [80], and integrative chemoproteomic pipelines such as STEP (single-cell target profiling) can infer cell-type-specific drug-protein engagement in tissues [81]. Collectively, these developments suggest that future advances in probe chemistry, imaging, and single-cell proteomics will increasingly enable activity mapping at the level of individual cells within intact microenvironments.

Beyond the residues discussed above, many amino acid residues still lack well-established ABPP strategies. Nonpolar side chains such as Ala, Val, Leu, and Ile are inherently unreactive, while residues with relatively weakly reactive groups, such as the hydroxyls of Ser and Thr and the amides of Asn and Gln, pose challenges for achieving selectivity. Nevertheless, these residues are functionally important: they can undergo post-translational modifications (PTMs) (e.g., H3Q5 mono-amination) [82] and play critical roles in disease-relevant mutations (e.g., KRas G12S) [83]. Developing ABPP approaches for these amino acids is therefore highly significant, and we anticipate that advances in bioorthogonal chemistry will open new avenues in this direction.

### 3.2. Design of Photoaffinity-Labelling (PAL) Probe

**Figure 9:**
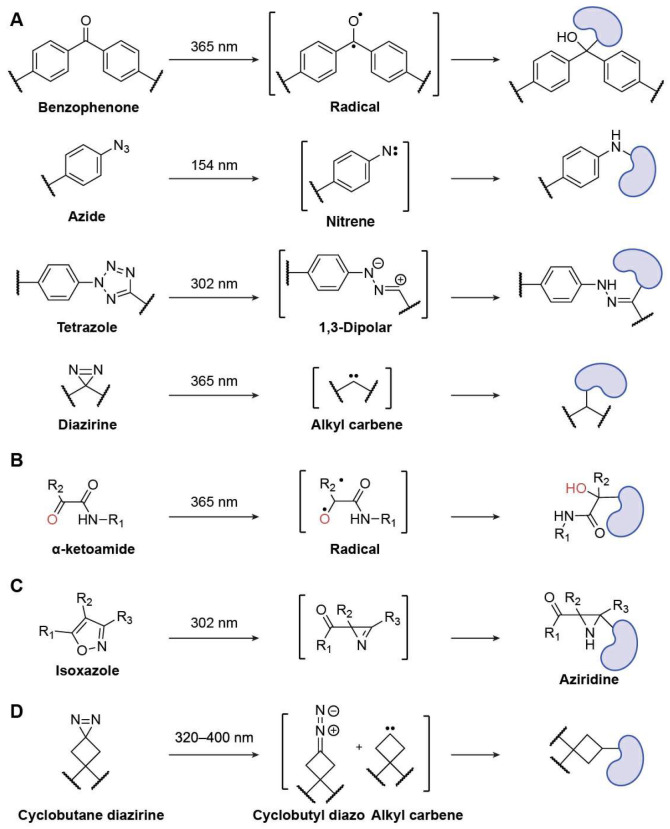
Representative PAL probes. (**A**) Benzophenone, Azide, Tetrazole, and Diazirine; (**B**) α-Ketoamide; (**C**) Isoxazole; (**D**) Cyclobutane diazirine.

PAL provides a means of investigating the interactions between proteins and ligands in the absence of a covalent warhead [9]. When exposed to ultraviolet (UV) light, the photo-crosslinking group produces a highly reactive intermediate that interacts with the molecule next to it to form a covalent bond between the target protein and the probe. Another name for this class of PAL-based probes is affinity-based probes (AfBPs). Among the different kinds of photo-crosslinkers, AfBPs most frequently use benzophenones, arylazides, tetrazole, and diazirines (Figure 9A) [84,85].

For photoreactive groups in PAL, 2-thienyl-substituted α-ketoamide is a superior option (Figure 9B). Research utilizing a variety of synthetic mannose-conjugated α-ketoamides showed that the 2-thienyl substitution of α-ketoamide lowered the rate of photodegradation and the electrophilicity of the keto group. Due to its decreased hydrophobicity, 2-thienyl ketoamide exhibited less labeling of nontarget proteins compared to representative conventional photoreactive groups [86].

Sun et al. developed a natively embedded photo-crosslinker for chemoproteomics using isoxazole, a typical pharmacophore that slightly changes a drug’s structure. Functionalized isoxazoles were developed and used for protein labeling, showcasing the increased photo-cross-linking effectiveness (Figure 9C). Subsequently, chemoproteomic investigations using two isoxazole-based medications, Danazol and Luminespib, revealed their potential cellular targets [87].

The invention of a cyclobutane diazirine photoaffinity tag with reduced pH-dependent reactivity, termed PALBOX, was reported by Christina M. Woo et al., who investigated the use of a ring strain to modify these reactivity preferences (Figure 9D) [88]. The findings demonstrate that PALBOX can be readily incorporated into small compounds to profile their binding interactions in cells and has distinct reactivity profiles in vitro when compared to other diazirine tags. Photoaffinity probes equipped with PALBOX can label known protein targets in cells with reduced labeling of known alkyl diazirine off-targets by using a set of small-molecule fragments and ligands. Additionally, the authors demonstrated how precisely ligands with PALBOX installed can map the binding sites between small molecules and proteins. Thus, PALBOX is a flexible diazirine-based photoaffinity tag that may be applied in the development of chemical probes for research on small-molecule-protein interactions as well as other photoaffinity-labeling applications.

## 4. Design of Cleavable Linkers

Within complex cellular lysates, various experimental objectives may call for linkers engineered to be cleaved under specific conditions. Beyond differences in cleavage efficiency, the most critical consideration is orthogonality. Common designs therefore favor simple, efficient reactions with minimal side effects, such as acid-base, redox, photochemical, or enzymatic cleavage strategies.

### 4.1. Acid/Base-Mediated Cleavable Linkers

**Figure 10:**
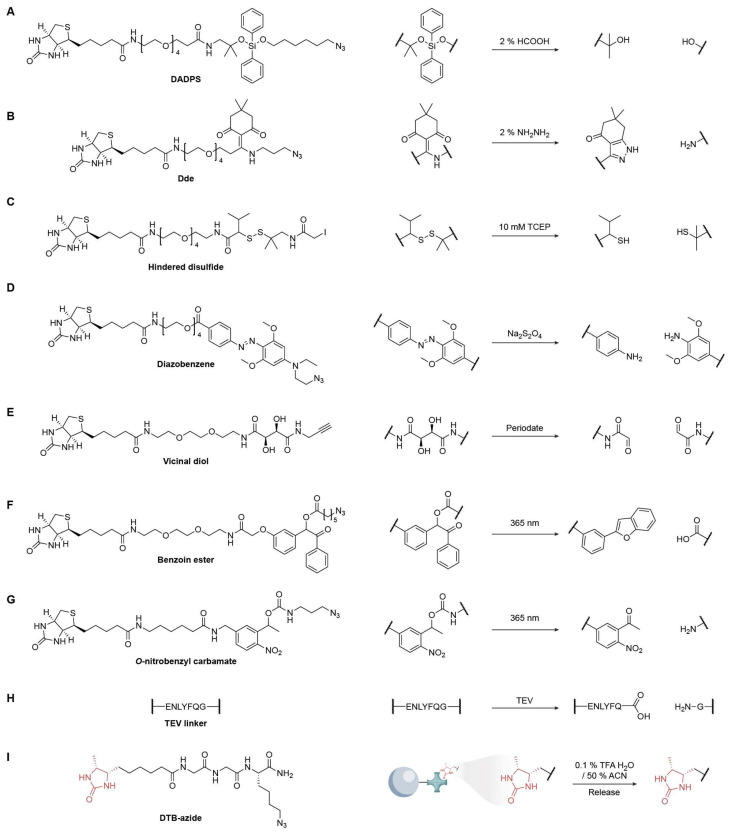
Representative cleavable linkers for chemoproteomic enrichment and release strategies. (**A**) DADPS; (**B**) Dde; (**C**) Hindered disulfide; (**D**) Diazobenzene; (**E**) Vicinal diol; (**F**) Benzoin ester; (**G**) o-Nitrobenzyl carbamate; (**H**) TEV linker; (**I**) DTB-azide.

Acid-labile linkers are widely used in chemical proteomics because peptides and proteins tolerate acid, enabling clean tag release after enrichment. DADPS linkers cleave efficiently under mild conditions (≈2% formic acid) and are available as cleavable biotin-azide tags for CuAAC capture of alkyne-labeled biomolecules (Figure 10A) [89]. A common issue is a +28.0 Da formylation artifact that occurs during formic acid cleavage and vacuum concentration. Yang and co-workers showed that lowering formic-acid strength and performing low-temperature speed-vac drying markedly suppress this side product [90]. The Verhelst group repurposed the peptide-synthesis-protecting group [1-(4,4-dimethyl-2,6-dioxocyclohex-1-ylidene)ethyl] (Dde) as a linker that is cleaved under very mild hydrazinolysis (≈2% hydrazine) (Figure 10B) [91]. Likewise, levulinoyl (Lev) linkers are stable in aqueous, acidic, and basic media, yet they are selectively removed by hydrazine, providing a robust alternative for activity-based protein profiling [92].

### 4.2. Reduction/Oxidation-Mediated Cleavable Linkers

Disulfides were among the first cleavable linkers because they fragment under mild reduction conditions, but they have two intrinsic drawbacks: (1) they are incompatible with reducing steps commonly used elsewhere in workflows (e.g., the Cu(I)-catalyzed azide-alkyne cycloaddition, typically generated with sodium ascorbate) [93], and (2) they undergo thiol–disulfide exchange with endogenous Cys/glutathione, leading to unexpected off-target release [94]. Sterically shielded disulfides mitigate exchange by installing bulky groups flanking the S-S bond [95]; these variants are relatively resistant to Dithiothreitol (DTT) yet remain cleavable by Tris (2-carboxyethyl)phosphine (TCEP), which attacks disulfides without forming mixed disulfides (Figure 10C).

Azobenzene linkers provide a complementary, reduction-cleavable handle (Figure 10D) [96]. The original Verhelst designs required higher concentrations of sodium dithionite (~25 mM), but tuning with electron-donating substituents on the rings accelerated cleavage to <10 s at ~1 mM, and further substitution enabled sub-second half-lives at ~0.5 mM, useful for rapid, benign release. Vicinal diol linkers enable oxidation-triggered scission with periodate (NaIO_4_, typically 1–10 mM) (Figure 10E) [97,98]. However, because periodate can oxidize cis-diols on glycans and other sensitive motifs, it would be prudent to develop more periodate-sensitive or orthogonally oxidation-labile motifs to enable lower oxidant dose and minimize undesired side reactions.

### 4.3. Photo-Irradiation-Mediated Cleavable Linkers

Photocleavable linkers provide reagent-free, spatiotemporally controlled release and are generally stable across acidic, basic, and redox conditions, failing only under specific irradiation. To avoid the biomolecular damage associated with deep UV (254 nm), benzoin ester linkers are designed to photolyze at 365 nm, completely liberating the tagged cargo (e.g., azidopentanoic acid) and forming a benzofuran byproduct in quantifiable yields (Figure 10F) [99]. Such linkers have enabled enrichment of proteins modified by lipid electrophiles like 4-hydroxynonenal (HNE). The Tirrell laboratory popularized *o*-nitrobenzyl (oNB) carbamate photocages, which are now among the most widely used photocleavable linkers owing to their excellent chemical robustness and efficient near-UV photolysis for selective release of captured proteins (Figure 10G) [89].

### 4.4. Chemoenzymatic Cleavage Linkers

In the TEV-cleavable biotin-azide tag, the azide and biotin modules are separated by a flexible linker bearing the seven-residue TEV recognition sequence ENLYFQ↓G (Figure 10H). After CuAAC labeling and streptavidin capture, on-bead TEV proteolysis releases the tagged peptides/proteins under near-neutral conditions, thereby bypassing the ultratight biotin–streptavidin interaction and avoiding harsh elution [4].

Aside from cleavage linkers enabled by bioorthogonal chemistry, desthiobiotin-azide (DTB-azide) offers an alternative approach. As a lower-affinity analog of biotin, it allows reversible streptavidin binding, with captured species eluted with mild acidic organic buffers (e.g., 0.1–1% TFA in 30–50% acetonitrile) or via competition with free biotin (Figure 10I) [100].

## 5. Discussion

This review details recent advances in ABPP techniques and the chemical design of ABPs. While ABPP methodologies are now well-established, ongoing innovations continue to emerge. For example, techniques such as SUPER-TOP ABPP, ABPP-CoDEL, and electroaffinity labeling [101,102,103] underscore the field’s evolution. Furthermore, significant improvements in ABP design have enabled the development of probes with enhanced labeling capacities and in vivo applications [104]. These advancements clearly demonstrate that while ABPP has reached a mature stage, it also holds tremendous potential for further innovation. From the perspective of bioorthogonal and bioconjugation chemistry, many potential reactions such as thiol radical generation and electron-triggered conjugation have not yet been explored as proteomic probes. The suitability of these warheads depends not only on their intrinsic activity, selectivity, and reaction kinetics but also on the stability of the resulting adducts under MS/MS conditions, which is essential for reliable proteomic analysis. Specifically, ABPP and ABPP-inspired chemistries have become powerful tools for PTM discovery and mapping, as PTM-directed probes selectively target the chemical functionalities introduced by these modifications. For example, multiple direct pull-down reagents targeting Cys S-sulfenylation (-SOH) have been developed and validated [105,106,107,108]. Additionally, metabolic chemical reporters embed bioorthogonal tags into PTMs in living systems, enabling site-resolved identification for protein modifications [109,110,111,112]. Furthermore, competitive ABPP provides an indirect readout of PTM occupancy: endogenous electrophiles or PTMs that pre-engage nucleophilic residues attenuate probe labeling, which can be quantified by quantitative ABPP to pinpoint hyper-reactive or PTM-sensitive sites proteome-wide [113]. Moreover, PTMs and protein isoforms can significantly influence ligandability, as these factors critically affect probe selectivity and biological interpretation. For example, changes in phosphorylation states [114] can remodel local binding pockets and alter covalent inhibitors’ engagement with oncogenic signaling proteins. An isoform-restricted allosteric Cys in JAK1 (C817) has been exploited to develop covalent inhibitors that preferentially label and inhibit JAK1 over other JAK isoforms [115].

Beyond experimental profiling, emerging artificial intelligence and machine learning (AI/ML) approaches are beginning to reshape covalent probe design and data interpretation. Sequence- and structure-based models can predict Cys reactivity and ligandability based on local environments and chemoproteomic measurements, providing complementary guidance to experimental screens [116], while dedicated covalent docking workflows enable evaluation of electrophilic warheads and reactive poses on structurally characterized targets [117]. Notably, AlphaFold2-based meta-analyses that integrate predicted side-chain accessibility with large Cys-profiling datasets have identified warhead-amenable sites across more than 95% of the human proteome, illustrating how AI/ML can support proteome-wide ligandability modeling and prioritize covalently targetable hotspots for future chemoproteomic studies [31]. Additionally, several web-accessible resources now allow researchers to explore and predict protein ligandability, such as CysDB [30], TopCysteineDB [118], and CovalentInDB 2.0 [119]. These tools offer practical entry points for interrogating ligandable hotspots in diverse biological contexts and underscore the growing intersection of chemoproteomics and machine learning, providing readers with actionable starting points for probe design and target prioritization. These strategic advances help not only discovery but also mechanistic linkage to function by integrating dose–response competition, stoichiometry estimates, and live-cell compatibility.

## 6. Conclusions

ABPP has evolved from a specialized enzyme-targeting method into a comprehensive chemoproteomic framework for mapping the reactive proteome, functionally annotating proteins, and guiding covalent drug discovery. Through continuous methodological innovation, the ABPP field now encompasses a diverse array of quantitative and site-resolved platforms, including TOP-ABPP, IsoTOP-ABPP, and competitive IsoTOP-ABPP, which collectively enable precise assessment of amino acid reactivity and small-molecule interactions at the proteome scale.

The rational design of reactive warheads has expanded ABPP’s reach beyond Cys to Lys, His, and other less nucleophilic residues such as Met, Tyr, and Trp, greatly broadening the chemical space of ligandable proteomes. In parallel, PAL probes have provided a powerful means of capturing transient or weak protein-ligand interactions through photoreactive groups such as diazirines, benzophenones, and isoxazoles, complementing PAL’s applicability to noncovalent-binding events. The incorporation of chemically and enzymatically cleavable linkers, including acid/base, redox, photo-irradiation, and protease-triggered versions, has further refined enrichment strategies, enhancing analytical depth, reproducibility, and orthogonality in downstream proteomic workflows.

Collectively, these advances highlight ABPP as a mature yet rapidly expanding discipline that integrates synthetic chemistry, mass spectrometry, and biological insights. Looking forward, the combination of ABPP with spatially resolved proteomics, single-cell technologies, and AI/ML-guided probe design promises to enable dynamic and predictive mapping of protein reactivity in living systems. Continued innovation in probe chemistry and analytical integration will further accelerate the discovery of functional residues and ligandable hotspots, advancing both basic chemical biology and translational covalent drug development.

## Data Availability

No new data were created or analyzed in this study. Data sharing is not applicable to this article.

## Abbreviations

The following abbreviations are used in this manuscript:

ABPP	Activity-Based Protein Profiling
ABP	Activity-Based Probe
AI/ML	Artificial Intelligence and Machine Learning
TOP-ABPP	Tandem Orthogonal Proteolysis–ABPP
IsoTOP-ABPP	Isotopic Tandem Orthogonal Proteolysis–ABPP
CuAAC	Copper-Catalyzed Azide–Alkyne Cycloaddition
TEV	Tobacco Etch Virus Protease
PAL	Photoaffinity Labeling
AfBP(s)	Affinity-Based Probe(s)
DADPS	Dialkoxydiphenylsilane
Dde	[1-(4,4-Dimethyl-2,6-dioxocyclohex-1-ylidene)ethyl] (Removable Linker)
Lev	Levulinoyl (Hydrazine-Cleavable Linker)
TCEP	Tris(2-carboxyethyl)phosphine
DTT	Dithiothreitol
oNB	o-Nitrobenzyl (photocage/linker)
HNE	4-Hydroxynonenal
ReACT	Redox-Activated Chemical Tagging
CuNiP	Cu(I)-Nitrene Platform
SuFEx	Sulfur Fluoride Exchange
SuTEx	Sulfur–Triazole Exchange
PPI	Protein–Protein Interaction
IA-alkyne	Iodoacetamide–alkyne
LDE(s)	Lipid-Derived Electrophile(s)
SDS-PAGE	Sodium Dodecyl Sulfate Polyacrylamide Gel Electrophoresis
TFA	Trifluoroacetic Acid
DMSO	Dimethyl Sulfoxide
PALBOX	Cyclobutane Diazirine Photoaffinity Tag
R (ratio)	Light/Heavy Intensity Ratio in IsoTOP-ABPP Quantification
